# Exploration of a New Source of Sustainable Nanomaterial from the *Koh-e-Suleiman* Mountain Range of Pakistan for Industrial Applications

**DOI:** 10.1038/s41598-020-57511-y

**Published:** 2020-01-17

**Authors:** J. S. Nirwan, S. Farhaj, M. M. Chaudhary, Z. Khizer, S. S. Hasan, A. Angelis-Dimakis, A. Gill, H. Rasheed, N. Abbas, M. S. Arshad, T. Hussain, Y. Shahzad, A. M. Yousaf, T. A. Chohan, T. Hussain, H. A. Merchant, M. R. Akram, T. M. Khan, M. Ashraf, B. R. Conway, M. U. Ghori

**Affiliations:** 1 Department of Pharmacy, School of Applied Sciences, University of Huddersfield, Huddersfield, HD1 3DH UK; 2 Lahore Waste Management Company, Lahore, Pakistan; 3 Department of Chemical Sciences, University of Huddersfield, Huddersfield, UK; 4 Ministry of Minerals and Mines, Lahore, Punjab Pakistan; 5 Pakistan Council of research for Water Resources (PCRWR), Ministry of Science and Technology, Islamabad, Pakistan; 6 University College of Pharmacy, the University of Punjab, Lahore, Pakistan; 7 Faculty of Pharmacy, Bahuddin Zakariya University Multan, Multan, Pakistan; 8 Department of Pharmacy, COMSAT University Islamabad, Lahore Campus, Lahore, Pakistan; 9 Institute of Pharmaceutical Sciences, University of Veterinary and Animal Sciences, Lahore, Pakistan; 10 The Wolfson Centre for Bulk Solid Handling Technology, University of Greenwich, London, UK; 11 System Engineering Department, Military Technological College, Muscat, Oman; 12 College of Pharmacy, University of Sargodha, Sargodha, 40100 Pakistan

**Keywords:** Environmental sciences, Environmental chemistry

## Abstract

The present study aimed to explore a new source of montmorillonite and to develop an extraction and purification protocol for its isolation from raw clay samples acquired from the *Koh-e-Suleiman* mountain range in Pakistan. The process involved the collection of raw clay from the source, identification and quantification of montmorillonite. Granulometric extraction and purification protocols increased the montmorillonite content from 21.8–25.1% in the raw clay to 90.1–93.9% after small-scale extraction and 85.33–89.33% on a larger scale. A techno-economic analysis highlighted the practicality and economic benefits of large-scale extraction for industrial applications. This study highlights the existence of a substantial new source of this valuable clay which is currently used across multiple industries including construction, pottery making, pharmaceuticals, cosmetics and engineering. It is intuitively expected that the large-scale extraction of the material will improve the economic condition of the region by providing employment opportunities to locals and may be a valuable resource for export.

## Introduction

The term clay generally refers to a natural material with plastic properties and particles or fragments of very fine size (<2 µm), composed mostly of hydrous-layer silicates of aluminium^1^. Based on chemical composition and atomic structure, clays can be classified into four groups: kaolinite, illite, chlorite and smectite^2^. As well as commonly known uses in construction and pottery making, their utilisation extends into multiple industries including pharmaceuticals, cosmetics, engineering and healthcare. The healing and medicinal properties of clays have long been recognised by humans and even animals, and other properties, such as high adsorption capacity, specific surface area, swelling capacity, reactivity to acids, dispersivity, hygroscopicity, unctuosity, thixotropy, plasticity and opacity, have led to their extensive and diverse range of applications^3–5^.

Montmorillonite clay has been identified as possessing desirable properties for exploitation across different industries, including pharmaceuticals, cosmetics, engineering and healthcare. It is a porous clay mineral belonging to the smectite group and is composed of a 2:1-layered structure with exchangeable cations between the layers^6^. It has been widely studied as an active ingredient in pharmaceuticals due to its swelling, rheological, moisture-retaining, adsorption, detoxification and anti-viral properties^7–9^. The adsorptive capacity of montmorillonite renders it suitable for drug entrapment and sustained-release, and its swelling and rheological properties may be used to optimise the physical and mechanical properties of formulations, including elasticity and tensile strength of gels or films^8^. Furthermore, its adsorption and healing properties have also led to its use in cosmetics as a natural remedy for oily skin and for the reduction of pimples^10^. Additionally, montmorillonite can adsorb toxic heavy metals and unwanted anions, such as fluoride in water, therefore, potentially being used as a material for water treatment as demonstrated by a number of studies^11–14^. Mohammed-Azizi *et al*.^13^ conducted a study on Algerian montmorillonite investigating the adsorption of aniline from an aqueous solution onto Algerian clay; results showed that the removal percentage of aniline by modified montmorillonite could reach up to 85%^13^. Keymirov 2018 also explored the ability of montmorillonite to remove ions of heavy metals, including Cu(II), Ni(II), Zn(II), Cd(II), from water and confirmed the efficacy of montmorillonite as a component in water treatment^14^. Other applications of montmorillonite are as in the oil drilling industry for maintaining the coolness of the drill and eradicating drilled solids, as well as a soil preservative to grasp soil water in drought-prone soils^2,15,16^.

The existence of major montmorillonite deposits has been indicated at various locations including the Himalayas in China, the Urals in north Pakistan, the Caucasus in Georgia and Russia, the Andes in Peru and Ecuador and the Wasatch mountains in Utah^2^. However, an abundant supply of raw clay also exists in a largely untouched mountainous location in South Punjab, Pakistan, the isolation, purification and characterisation of montmorillonite from these raw clays was unexplored. Due to large dependence on agriculture and low levels of industrialisation, South Punjab currently faces higher levels of poverty and unemployment compared with the rest of the province^17^. Hence, it is expected that harvesting this valuable resource may provide economic benefits for the region. However, these materials may contain a number of undesirable substances including lead, arsenic and crystalline silica which may not only impede their industrial potential but may also have adverse health effects if employed in raw form^18^. To meet reproducibility, content and purity standards set by regulatory agencies, it is crucial that these clays undergo rigorous purification for the standardisation of their physicochemical and functional properties.

**Figure 1:**
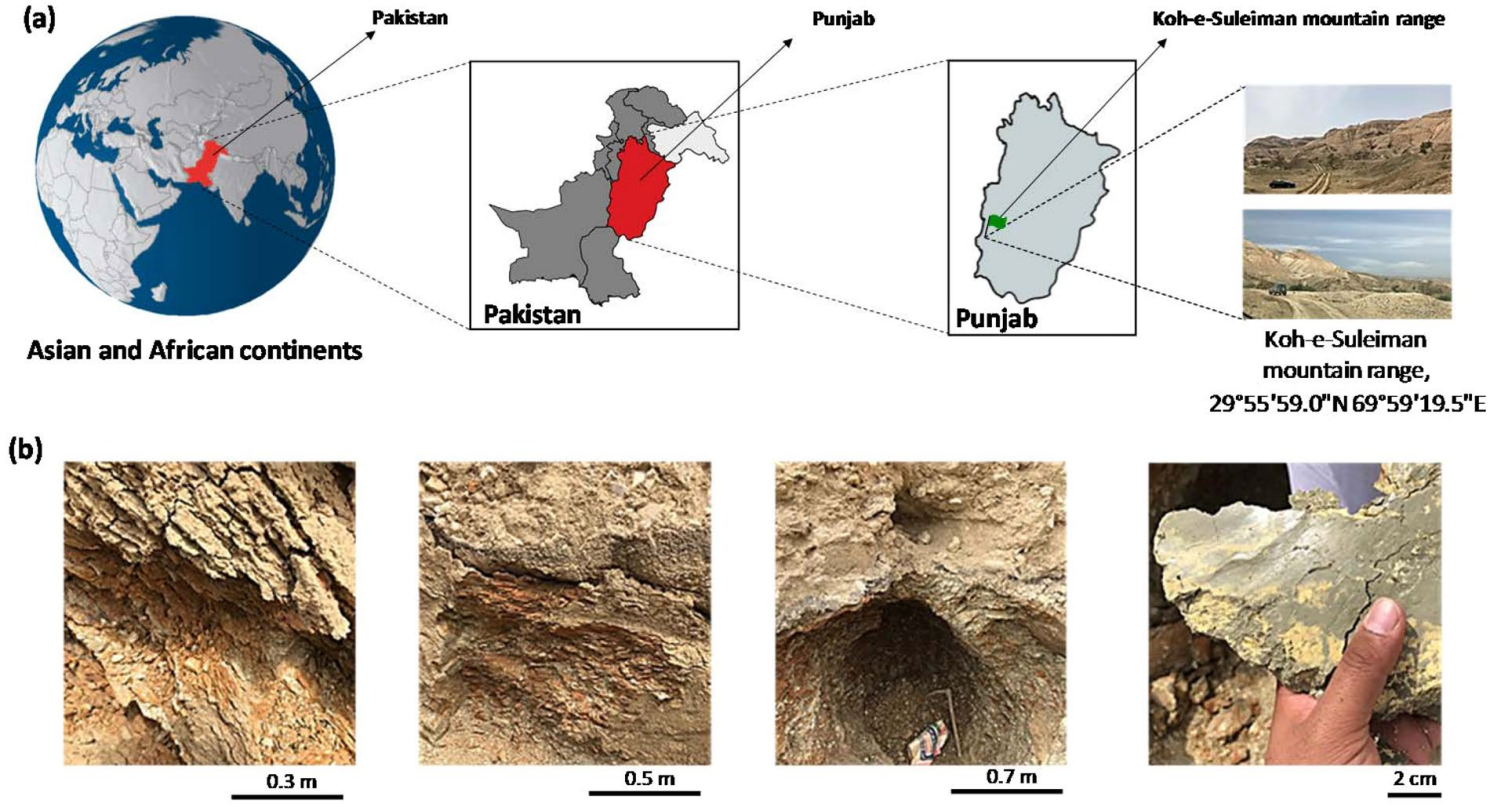
(**a**) Geological map of Pakistan and (**b**) site for raw clay sample collection.

Therefore, the aim of this study was to develop a granulometric purification protocol from small to large scale for the isolation of montmorillonite from raw clay samples obtained from the *Koh-e-Suleiman* mountain range of Pakistan (Fig. 1). Characterisation of the purified montmorillonite samples was carried out including the techno-economical cost estimated to establish its suitability for use in industrial applications.

## Results and Discussion

### Identification of montmorillonite

**Figure 2:**
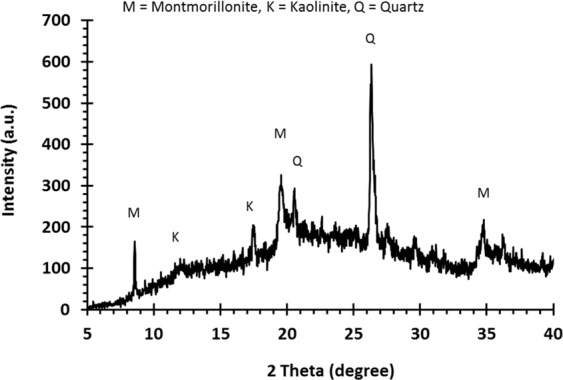
XRD patterns of raw clay.

The samples of raw clay obtained from the *Koh-e-Suleiman* mountain range were subjected to XRD analysis which displayed the characteristic peaks indicating the presence of montmorillonite in the raw clay samples along with a significant amount of impurities, namely quartz and kaolinite (Fig. 2). Further analysis using the Cu-TET method revealed the proportion of montmorillonite present in the samples ranged from 21.79 ± 3.21% (obtained using XRD) and 25.11 ± 1.87% (using a Cu-TET method), although these differences were statistically insignificant. Although these data indicate a considerable amount of montmorillonite in the raw clay, levels were substantially lower than the proportion of the mineral present in raw clays in other locations^18–20^. For example, a sample of raw bentonite collected from the mining site of Anji Gaoyu, Zhejiang province in China comprised 44% montmorillonite, while raw clay collected from a Pakistani mining site of Khyber Pakhtunkhwa province located in Shagia contained 75% montmorillonite^18,19^.

**Figure 3:**
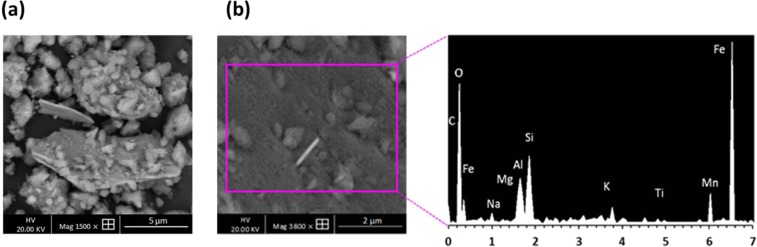
(**a**) SEM micrographs and (**b**) EDS spectra of raw clay.

Further analysis of the clay by SEM revealed heterogranular agglomerated particles of approximately 12 µm in length (Fig. 3a). Elemental composition by SEM/EDX (Fig. 3b) determined the proportions of the main components constituting the clay minerals. The largest component was oxygen followed by iron (Fe) (18.21%), silicon (Si) (17.21%) and aluminium (Al) (12.1%). Small proportions of magnesium (Mg) (3.21%), sodium (Na) (2.90%), potassium (K) (2.80%), and calcium (Ca) (1.95%) were also present in the clay (Fig. S1). These results are characteristic of montmorillonite which has the chemical formula (Na, Ca)_0.33_ (Al, Mg)_2_Si_4_O_10_(OH)_2_.n(H_2_O). The basic structural unit of montmorillonite mineral consists of one alumina octahedral sheet sandwiched between two tetrahedral silica sheets. Due to the isomorphic substitution of Al^3+^ and Fe^3+^ ions by Mg^2+^ and Fe^2+^ ions in the octahedral sheet and Si^4+^ by Al^3+^ and Fe^3+^ ions in the tetrahedral sheets, each unit has excess negative permanent charge at its basal surface. However, most of the substitutions occur in the octahedral sheet and the negative surface charge is compensated by adsorbed cations such as Na^+^, K^+^, Ca^2+^ or Mg^2+^^21,22^.

### Optimisation of extraction/purification process

**Figure 4:**
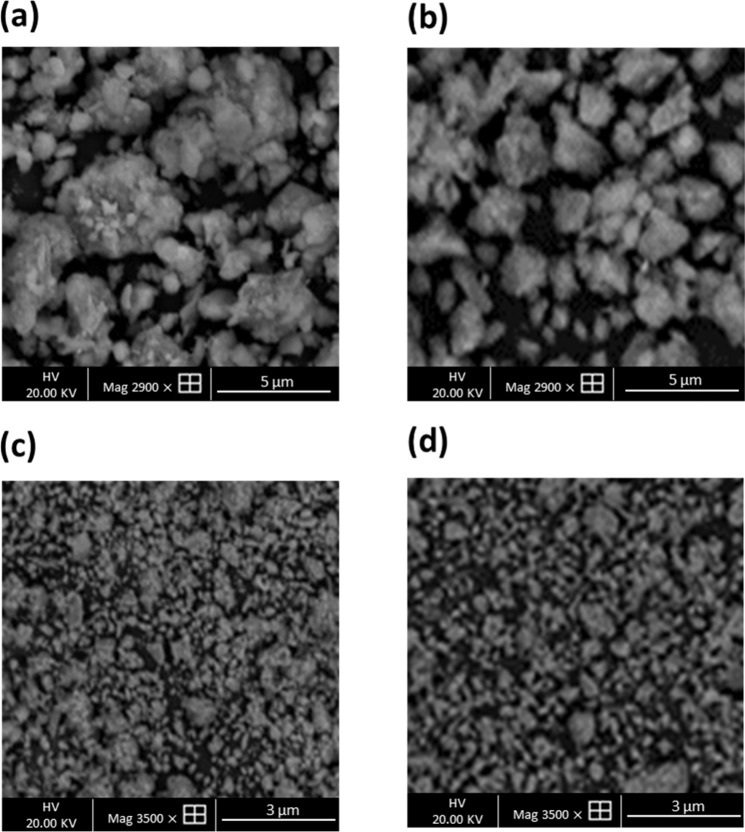
SEM micrographs of montmorillonite particles ground for (**a**) 20 min, (**b**) 40 min, (**c**) 60 min and (**d**) 80 min.

**Figure 5:**
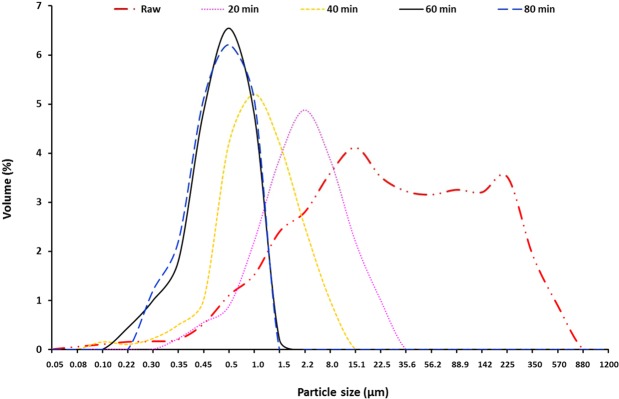
Particle size distribution of montmorillonite particles ground for (**a**) 0 min, (**b**) 20 min, (**c**) 40 min, (**d**) 60 min and (**f**) 80 min.

Following the identification of montmorillonite in the raw clay, the sample was ground for different durations (20, 40, 60 and 80 min) and then analysed to determine the optimal grinding time for the extraction process. SEM images collected at each time point are displayed in Fig. 4a–d. After 20 min of grinding, the SEM image shows large particles and substantial heterogeneity in the shapes and sizes of the particles (Fig. 4a). This was also reflected in the analysis of particle size distribution displayed in Fig. 5 which ranged from 0.30–35.6 µm with a mean diameter of 2.75 µm. Comparatively, particle size analysis of the raw clay displayed a broad distribution in particle size with an asymmetric tail and 2 maxima (15.1 µm and 225 µm) with a mean diameter of 9.15 µm. Grinding the raw clay for 40 min reduced the mean diameter to 1.1 µm with a range of 0.08–15.1 µm. The SEM image displayed in Fig. 4b also showed increased homogeneity in particle size and shape. This trend was also visible in the SEM analysis of the clay after 60 min of grinding and particles were relatively homogeneous is size (Fig. 4c).Particle size distribution was 0.10–1.75 µm following 60 min grinding and 0.22–1.5 µm following 80 min grinding and SEM images were similar. The mean diameters (*D*_50_) for clay ground for 60 min and 80 min were 0.51 µm and 0.48 µm, respectively. These values are ideal for montmorillonite liberation and are in accordance with literature where the particle size of montmorillonite is usually between 0.1 μm and 2 μm with an average diameter of ~0.5 μm^23^.

EDX analysis of the raw clay at each time point is displayed in Figure S2a–d. This data revealed a considerable decrease in the proportion of impurities (Na, Mg, Al, K, Ca and Fe) in the clay compared with the raw clay, while the proportion of Si increased. This could be seen in the elemental analysis which also displayed a significant decrease in mass (%) of Fe and Al from 20 min to 60 min and a significant increase in silicon (Fig. S3a–d). However, there were no further changes between 60 min and 80 min grinding duration (Fig. S3c,d).

**Figure 6:**
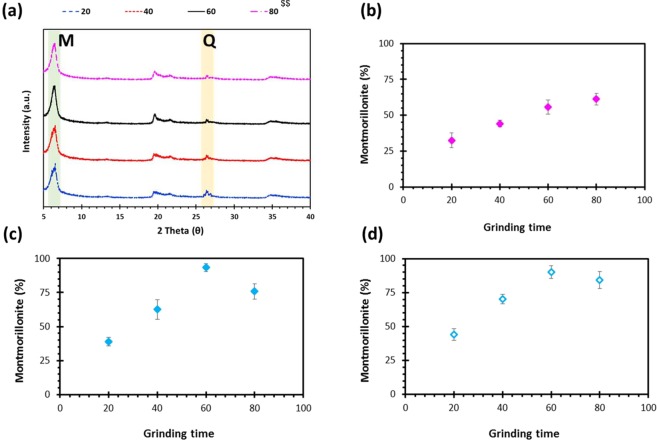
(**a**) XRD, (**b**) extracted yield and (**c**,**d**) content, using (**c**) XRD and (**d**) Cu-TET methods, of montmorillonite clay particles (^$$^showing different grinding time).

XRD (Fig. 6a) showed an increase in peak intensity for montmorillonite (M) and a decrease in peak intensity for quartz (Q) from 20 min to 60 min grinding time. This was also reflected in Fig. 6c,d which displays the peak intensity ratio of montmorillonite relative to that of montmorillonite plus quartz versus grinding time obtained using XRD (Fig. 6c) and Cu-TET (Fig. 6d). After 20 min of grinding, the montmorillonite content was 38.96 ± 3.22% according to the XRD method and 44.25 ± 4.26% using the Cu-TET method. Grinding for 40 min increased the montmorillonite further up to 62.67 ± 7.23% and 70.16 ± 3.55% using XRD and Cu-TET methods, respectively. The montmorillonite content reached a peak after 60 min of grinding (93.95 ± 2.88% and 90.11 ± 4.53% using XRD and Cu-TET, respectively) and increasing grinding time to 80 min decreased montmorillonite content (75.89 ± 5.66% and 84.23 ± 6.31% using XRD and Cu-TET, respectively). This is in contrast to the mass product yield shown in Fig. 6b. Therefore, the reduction in relative montmorillonite content from 60 min to 80 min displayed in Fig. 6c,d may be due to an increase in the content of quartz as previously observed by Gong *et al*.^19^.

**Figure 7:**
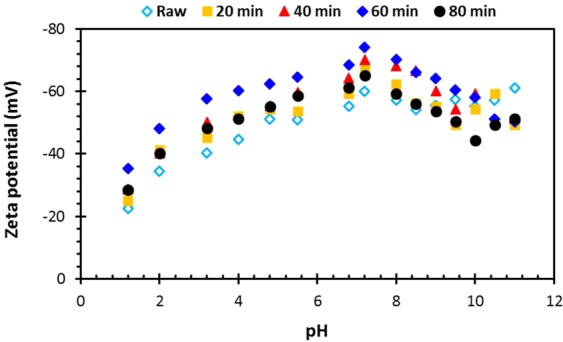
Zeta potential charge of clay particles as a function of pH.

The zeta potential of clay and the impact of grinding was measured over a pH range from 1.2 to 11 (Fig. 7). The raw clay and ground clay ground for all time points followed the same trend. At pH 2, the zeta potential was least negative (raw = −22.5 mV, 20 min = −25.1 mV, 40 min = −28.7 mV, 60 min = −35.4 mV, 80 min = −28.6 mV) and became more negative as the pH was increased up until pH 7.2 (raw = −60.12, 20 min = −67.3 mV, 40 min = −70.2 mV, 60 min = −74.3 mV, 80 min = −65.3 mV). As the pH was further increased to 10, the zeta potential became less negative. This behaviour is due to dissolution of the edges of the montmorillonite structure at extreme values of pH, which leads to breaking of the bridge oxygen bond (Si–O–Al), releasing aluminium and then silicon atoms into solution. At acidic pHs, this process results in the release of Al^3+^ which displaces the naturally occurring sodium, resulting in a less negative zeta potential. At a higher pH, dissolution results in the release of Al(OH)_4_^-^ which is repelled from the negatively charged particle surface due to electrostatic repulsion. At these high values, Na^+^ is the main cation which is weakly bound and results in a less positive charge being retained within the shear plane, and a negative zeta potential over pH values in the basic range^24^. Additionally, the results displayed the highest zeta potential for clay particles ground for 60 min and the lowest for the raw clay. As zeta potential reveals the extent of electrostatic repulsion between similarly charged particles located adjacently, a high zeta potential indicates greater stability i.e., the solution or dispersion will resist aggregation^21,25^.

Multiple studies have found that the value of CEC is proportional to the clay content in the sample^20,26–28^. In this study, the CEC ranged from 0.88 to 0.99 meq/g in purified samples, (Table 1), however, the raw sample displayed the lowest CEC (0.79 meq/g). It is also evident from the results that the CEC increased as grinding time increased. Furthermore, the CEC values were in the range characteristic to clays in the smectite group (0.76 to 1.5 meq/g)^28–33^. Table 1 also displays the results from nitrogen adsorption-desorption studies which applied the Brunauer, Emmett and Teller (BET) method and Barrett Joyner-Halenda (BJH) model to assess the specific surface area and pore size distribution, respectively. As expected, there was increase in both specific surface area and external surface area and porosity (micropore area, total volume and micropore volume) with grinding duration due to the decrease in particle size. However, these properties decreased from 60 min to 80 min possibly due to agglomeration of particles.

**Table 1:** CEC and nitrogen adsorption parameters for raw and purified montmorillonite clay samples.

Clay samples		CEC (meq/g)*		Parameters of nitrogen adsorption studies				
				*SS*_*A*_ *m*^2^/g**	*ES*_*A*_ *m*^2^/g***	*A*_*m*_ *m*^2^*/g*^$^	*V*_*T*_ *cm*^3^*/g*^$$^	*V*_*m*_ *cm*^3^*/g*^$$$^
Raw		0.79		55.41	35.39	28.64	0.055	0.010
Purified (small scale)	Grinding time	20 min	0.88	78.22	43.16	33.67	0.071	0.014
		40 min	0.94	98.15	54.94	42.55	0.085	0.019
		60 min	0.99	104.33	59.11	46.83	0.092	0.022
		80 min	0.98	99.28	56.32	45.17	0.088	0.020
Purified (Large scale)		0.98		100.29	58.19	45.99	0.086	0.020

^*^CEC, cation exchange capacity; ^**^SSA, specific surface area; ^***^ESA, external surface area, ^$^Am, micropore area; ^$$^VT, total volume; ^$$$^Vm, micropore volume.

No pathogenic bacteria were detected in the samples and the total bacterial count was below the total aerobic acceptance limit for clays set by the US Pharmacopoeia and no contamination by *E. coli* was observed^34^. To allow efficient extraction of montmorillonite from the raw clay, an appropriate grinding intensity and time is essential. For this to be effective, it is essential that the particle size of the montmorillonite is small enough to enable it to be released from mixed aggregates. Excessive grinding, however, can produce an ultrafine particle suspension with a dispersion difference between montmorillonite and associated minerals which is not sufficient to allow effective separation. Therefore, it is necessary to determine the optimal grinding time which, in this study, was concluded to be 60 min^19^.

### Small-scale vs large-scale extraction

**Figure 8:**
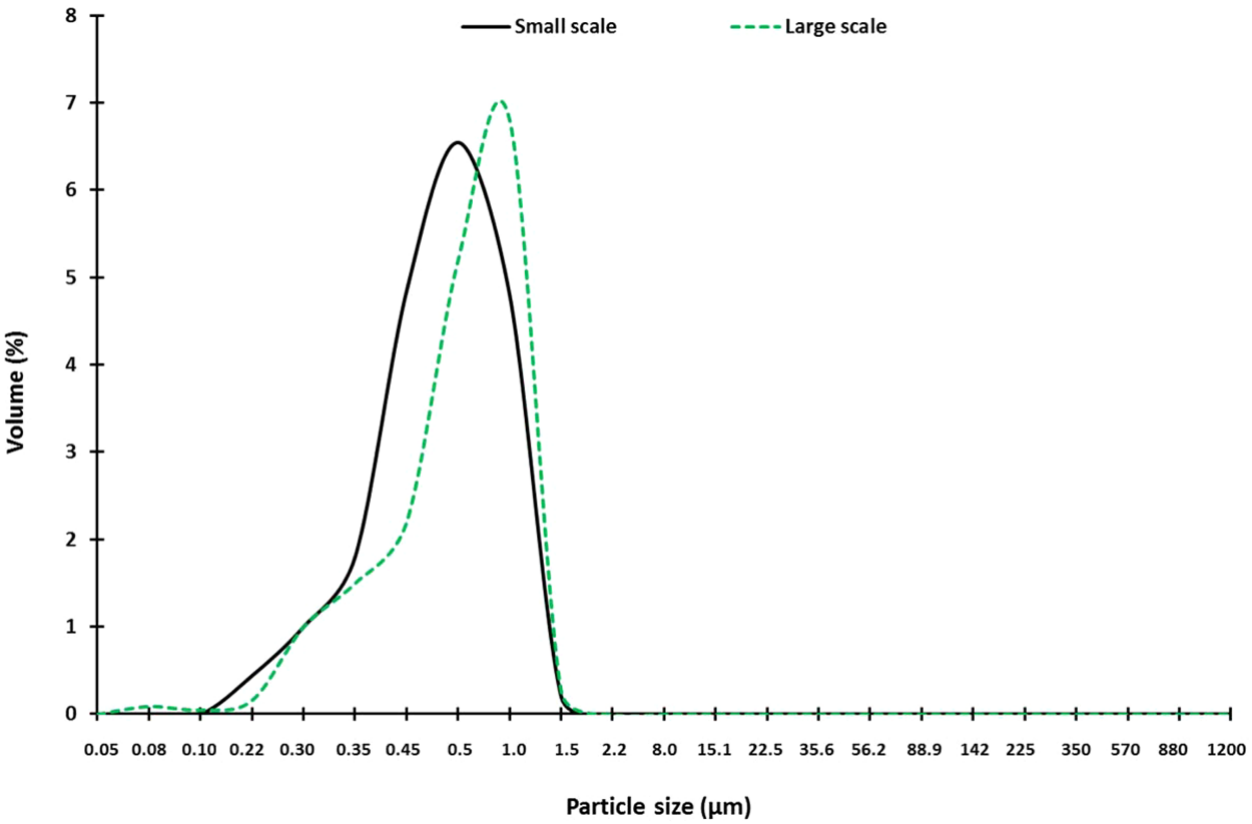
Particle size distribution of montmorillonite particles extracted on small and large scales.

Having established the suitability of the extraction process in producing montmorillonite suitable for industrial applications, the procedure was scaled-up. An SEM micrograph of the clay produced on a larger scale is shown in Fig. S4a with similar, albeit, slightly larger plate-like particles compared with the small-scale extracted clay. Comparing the EDX analysis and atomic distribution after small and large-scale extraction demonstrates a substantial increase in weight (%) of Si (large-scale = 36.2 ± 2.5 *vs* small scale = 33.6 ± 6.2) and reduction in impurities (Fig. S4a–c). The particle size distribution of small-scale and large-scale extracted clay is displayed in Fig. 8. Particle size range of large-scale extracted clay was 0.05–1.91 µm in comparison with the small scale extracted montmorillonite, 0.10–1.72 µm. Moreover, the mean diameter, *D*_50_, was 0.62 µm and 0.51 µm for large and small scale extracted montmorillonite, respectively. The observed particle size during montmorillonite extraction scale up are acceptable, and are in accordance with literature where the particle size of montmorillonite is usually between 0.1 μm and 2 μm with an average diameter of ~0.5 μm^23^.

**Figure 9:**
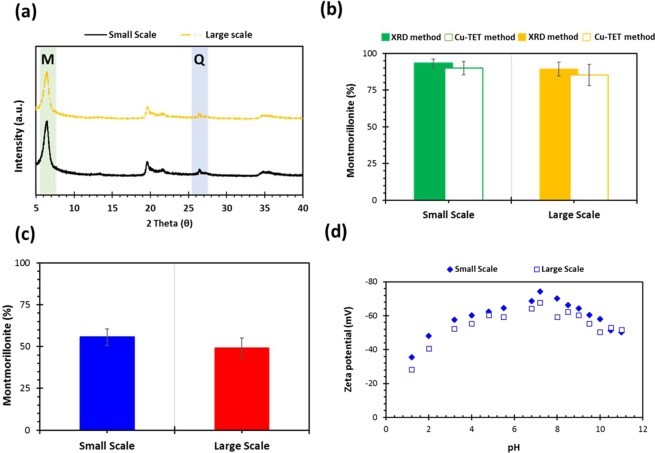
(**a**) XRD, (**b**) content, (**c**) extracted yield and (**d**) zeta potential charge of montmorillonite clay particles with a function of pH.

Analysis of small-scale and large-scale extracted clay using XRD produced peak patterns associated with montmorillonite as evident in the literature, Fig. 9a^18,19^. Upon visual analysis, the small-scale extracted clay has a montmorillonite peak with marginally higher intensity than the peak attained by the large-scale extracted clay. Figure 9b showed montmorillonite content in small-scale extracted clay (93.95% and 90.11% using XRD and Cu-TET, respectively) compared with large-scale extracted clay (89.33% and 85.33% using XRD and Cu-TET, respectively). Figure 9c exhibited montmorillonite yield obtained during the small and large scale extraction (small-scale 55.69% *vs* small-scale 49.11%). Figure 9d displays the zeta potential of small-scale and large-scale extracted clay measured over a pH range from 1.2 to 11. Similar to different small-scale ground clay fractions, the zeta potential was least negative at pH 2 (small-scale = −35.39 mV; large-scale = −28.33 mV) and became more negative as the pH was increased up until pH 7.2 (small-scale = −74.22 mV; large-scale = −67.59 mV). As the pH was further increased, the zeta potential became less negative up until pH 10. On comparison, all the characteristic attributes (elemental analysis, montmorillonite content and yield, particle size, nitrogen adsorption parameters and zeta potential charging) of small and large scale extracted montmorillonite clay samples have shown statistically insignificant differences. Moreover, microbiological tests displayed no pathogenic bacteria in the large-scale extracted clay as well as no contamination by *E. coli*. The total amount of bacteria was also within the total aerobic acceptance limit set by the US Pharmacopoeia. Hence, all these findings are highlighting the suitability of the large-scale extraction protocol for producing clays appropriate for pharmaceutical, biomedical, healthcare and environmental applications^34^.

### Techno-economical estimation of purified montmorillonite

**Figure 10:**
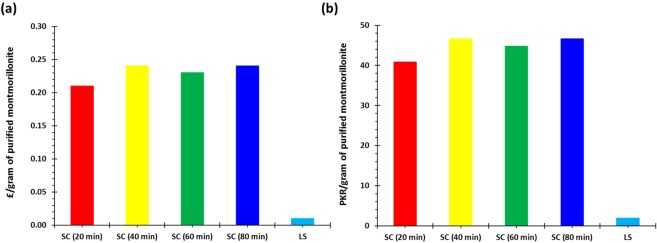
Total cost of associated with montmorillonite clay purification in (**a**) UK currency and (**b**) Pakistani currency.

**Figure 11:**
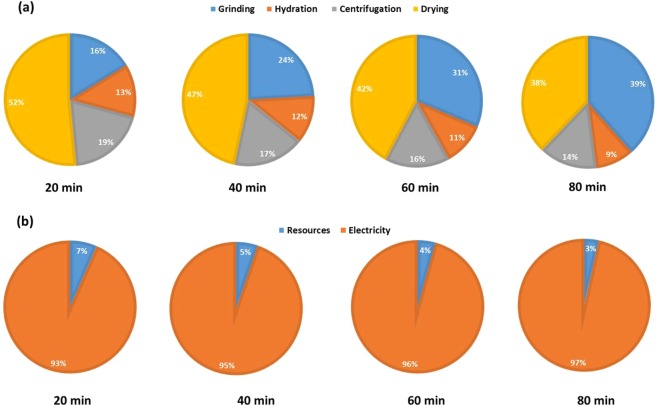
Costing associated with montmorillonite clay purification with respect to grinding time (**a**) per stage and (**b**) per component breakdown.

**Figure 12:**
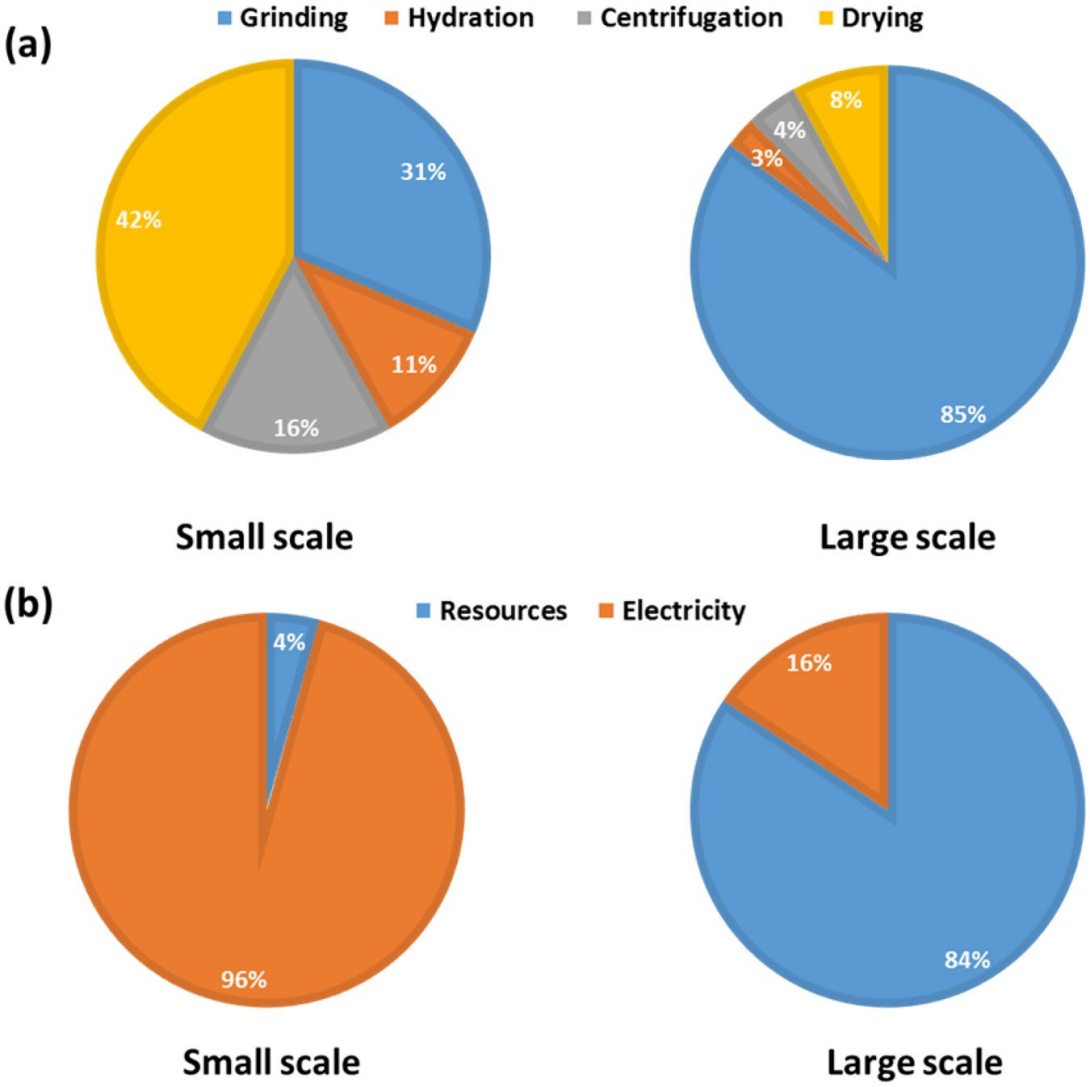
Costing associated with montmorillonite clay purification at small and large scale (**a**) per stage and (**b**) per component breakdown.

To investigate the practicality and economic benefits of large-scale extraction and purification of montmorillonite from the new source for industrial applications, a techno-economic analysis was conducted. Figure 10 displays the estimated costs per gram of extracting and purifying montmorillonite on both scales in UK currency (£) and Pakistani currency (PKR). This revealed an increase in the costs involved in small-scale extraction from 0.21 to 0.24 £/g and from 40.8 to 46.6 PKR/g as the grinding time was increased from 20 min to 40 min. Conversely, the costs for small-scale extraction using a grinding time of 60 min (0.23 £/g and 44.7 PKR/g) was lower than 40 min. Moreover, the costs for extraction using a grinding time of 80 min (0.24 £/g and 46.6 PKR/g) was equal to that of 40 min. As the grinding time increases and the other processes (hydration, centrifugation and drying) remain consistent and unaltered, the proportion of the total costs consumed by the grinding process and by electricity also increases as shown in Fig. 11a,b. This also explains the increase in costs per gram from 20 min to 40 min grinding time as the increase in yield from 1.95 g to 2.15 g was insufficient to compensate for the increase in costs of grinding. The increase in yield from 40 min to 60 min was greater (2.15 g to 2.74 g) which led to a decrease in costs per gram, whereas the increase in yield from 60 min to 80 min (2.74 g to 3.19 g) was again inadequate to prevent an increase in cost. However, the cost per gram of large-scale extraction of montmorillonite was considerably lower than small-scale (0.01 £/g and 1.9 PKR/g). Although the proportion of the costs consumed by the grinding process was substantial (85%) (Fig. 12a) mainly due to a large increase in the resources required (namely sodium triphosphate) (Fig. 12b), the yield obtained from the extraction (50.26%) was significant enough to offset this increase.

## Conclusions

This study successfully identified and extracted montmorillonite from a new source in the *Koh-e-Suleiman* mountain range of Pakistan. A granulometric extraction and purification protocol was successfully developed for the small-scale and large-scale extraction and purification of the clay allowing the montmorillonite content to be increased from 21.8–25.1% in the raw clay to 90.1–93.9% in the small-scale extracted clay and 85.4–89.4% in the large scale extracted clay. Techno-economic analysis of small-scale and large-scale extraction also revealed the economic benefits of extracting the clay on a large-scale. Microbiological and physicochemical analysis confirmed the suitability of the large-scale extraction protocol for producing clays appropriate for pharmaceutical, environmental, healthcare, cosmetic and biomedical applications. Hence, it is anticipated that highlighting the existence of a substantial new source of this valuable clay will attract the attention of multiple industries and will lead to large scale extraction of the material. It is expected that this will improve the economic condition of the region by providing employment opportunities to locals and a valuable resource for exportation.

## Materials and Methods

### Materials

Raw clay was obtained from the *Koh-e-Suleiman* mountain range located in the Southern region of the Punjab province, Pakistan (29°55′59.0″N 69°59′19.5″E) (Fig. 1). Ten samples (5 kg each) were collected using a hoe and shovel, packed in plastic bags with air tight seals and dried at 105 °C until reaching a constant weight. They were then mechanically ground using a ball mill. Equal proportions of bulk samples (1 kg) were mixed and homogenized to form a single representative sample that was used for all further investigations.

### Methods

**Figure 13:**
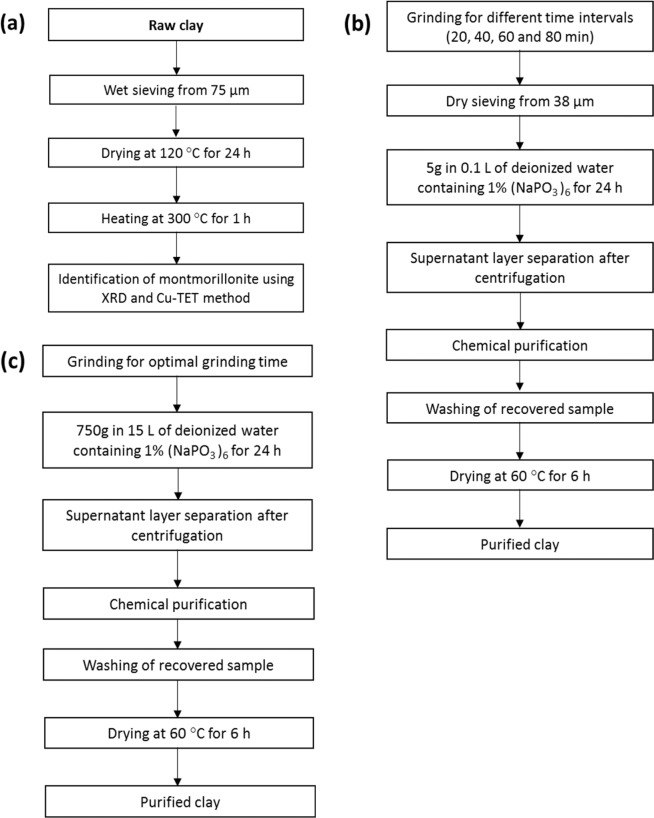
Flowchart detailing the protocol for (**a**) identification, (**b)** small scale and (**c**) large scale extraction of montmorillonite.

A flowchart depicting the protocol for identification, small-scale and large-scale extraction of montmorillonite is presented in Fig. 13a–c.

#### Identification of montmorillonite in raw clay

Raw clay samples were wet sieved using a 75 µm mesh screen and dried at 120 °C for 24 hours. The dried samples were heated at 300 °C for 1 hour and montmorillonite content was determined using X-ray diffraction (XRD) and Cu (II) – triethylenetetramine complex (Cu-TET) adsorption methods. Scanning electron microscopy with energy dispersive X-ray spectroscopy (SEM/EDX) was used for morphology and elemental analysis.

#### Small scale granulometric purification of raw clay

Following identification of montmorillonite, raw clay was ground using ball mill (Mixer Mill MM 200 by Retsch Ltd. Hope, UK) for different durations (20, 40, 60 and 80 min) to determine the optimal grinding time. Cu-TET adsorption technique was used to determine the montmorillonite content. Using XRD, the peak intensity for montmorillonite compared with impurities was assessed. The time point at which maximum montmorillonite content was determined and the greatest peak intensity for montmorillonite and the lowest peak intensities for impurities were achieved, was concluded as being the optimal grinding time.

Raw clay hydration and centrifugation. Raw clay (5 g) was immersed in 1 L of deionized water, agitated for 24 h with 1% w/v of dispersant ((NaPO_3_)_6_), centrifuged for 8 min at 1000 rpm and the fine fraction in dispersion was collected. The solid particle shape was assumed to be spherical and Stokes’ law was used to estimate the centrifugation time: Eq. 1^20^

1 $${\rm{t}}=[\eta \,{\log }_{10}\,({\rm{R}}/{\rm{S}})]/[3.81\,{{\rm{N}}}^{2}{{\rm{r}}}^{2}\Delta {\rm{S}}]$$

where,

t = centrifugation time (second)

R = distance from the deposit surface to the axe of rotor (14 cm)

S = distance from the suspension surface to the axe of rotor (4.2 cm)

N = rotation speed = (1000 rpm)

r = maximum radius in cm of the desired particles (cm) = (2·10^−5^ cm)

ΔS = specific gravity difference between the particles and the liquid suspension (0.00528 g cm^−3^)

η = viscosity of the fluid (0.00748 poise at 25 °C)

Following centrifugation, all traces of the dispersant were removed from each size-fraction by washing with distilled water. Each fraction was exposed to the centrifugation process at least three times successively until the mass ratio of the dispersed particles collected in the solution with respect to the total amount of pristine material (i.e. 5 g) was negligible.

Chemical purification. Classical purification methods were employed to remove the carbonates, humic acids and iron oxides adsorbed onto the clays^35–41^. The carbonates were removed using a decomposition method, briefly, 5 g of clay, after hydration and centrifugation stages, was dispersed in 100 mL of 1 mol L^−1^ acetic acid solution and the pH of this solution was buffered to 4.8 using 0.1 mol L^−1^ sodium acetate, which was introduced into the dispersion dropwise with continuous monitoring. This slurry was then left for 4 hours until carbon dioxide production ceased. The solid phase was then separated using centrifugation at 4400 rpm for 15 min and dried at 80 °C for 4 h. After drying, organic material was removed. Sodium acetate (250 mL; 0.1 M) and 80 mL of a 30 mass % hydrogen peroxide solution were added to 5 g of dried clay sample. The slurry was stirred for 6 h at 80 °C and then for 2 h at room temperature, and the solid phase was separated using centrifugation at 4400 rpm for 15 min and dried at 80 °C for 4 h. Further, the iron oxide was removed using the method described elsewhere^35,37,42,43^. The resultant clay samples were centrifuged and washed for a minimum of two times with a 0.5 mol L^−1^ sodium chloride solution. Finally, the clay was washed three times in distilled water to eliminate the residual NaCl and then dried for 6 hours at 60 °C and purified clay was obtained.

#### Large scale granulometric purification of raw clay

Raw clay was ground using a commercial grain grinder for the optimal grinding time, 60 min. Ground clay (750 g) was n soaked for 24 hours in 15 L of deionised water containing 1% w/v (NaPO_3_)_6_ using large bespoke glass vessel of 20 L capacity and then centrifuged for 8 min at 1000 rpm. The supernatant layer was separated, washed and then dried for 6 hours at 80 °C and the dried clay sample was then subjected to chemical purification and washing, described in section 4.2.2.2. The samples were then dried for 6 hours at 60 °C to obtain a purified clay.

#### Characterisation of raw and purified montmorillonite clay

X-ray diffraction (XRD).Raw clay samples were characterised using X-ray powder diffraction using a Bruker D2 Phaser XRD diffractometer. XRD patterns were collected from 2θ = 2° to 50° by step of 0.05°, 12 s per step. Oriented samples were obtained by deposition of raw clay dispersions on glass slides. The crystallite size was calculated by the Scherrer formula taking into account the instrumental broadening.

Cu (II) – triethylenetetramine complex (Cu-TET) adsorption method. This method is based on measuring adsorption of a copper complex onto montmorillonite particles contained in the raw clay^44^. A weighed amount of raw clay was mixed in water (ratio of 1:20) using ultrasound. Cu(II)-triethylenetetramine solution (10 ml; 0.1 M) was then added and topped-up with water until a total volume of 50 ml was reached. Following this, the mixture was centrifuged and the clear supernatant layer was removed. Spectrophotometric determination was performed at a wavelength of 620 nm. The adsorbed amount of Cu (II) complex was determined from the measurement difference against water which was used to calculate the percentage of montmorillonite content in raw clay.

Scanning electron microscopy with energy dispersive X-ray spectroscopy (SEM/EDX). Briefly, the powdered sample was mounted on a metal stub using double-sided adhesive tape. The sample was then sputtered coated with gold/palladium (80:20) for 60 seconds and samples were examined using a Quanta FEG 250 environmental microscope (Thermo Fisher Scientific, Massachusetts, USA).

For localised chemical analysis, a Kevex Sigma energy dispersive spectrometer, X-ray spectrometer, coupled to the microscope was used. The quantitative analysis of samples was executed at accelerating voltages of 15–50 kV, and at a detection time of 150 s with an X-ray fluorescence element analyser.

Nitrogen adsorption. The samples were characterized by nitrogen adsorption at T = 77 K using a Micromeritics Tristar 3000 apparatus. Specific surface areas, microporous area and pore size distribution were estimated by the BET model, the t-plot method and the BJH model^45^.

Particle size analysis. A Mastersizer S granulometer was used for the determination of particle size distribution (size range: 0.05–900 μm). Before measurements, particles were dispersed in water and sonicated.

Cation exchange capacity (CEC). Each sample (5 g) was added to 200 mL of 3 M ammonium acetate solution at pH 7.2, stirred magnetically for 12 h and placed undisturbed overnight^46^. Following this, the sedimented clay samples were washed with ethanol three times and dried at 60 °C for 4 h. CEC was determined using a Kjeldahl Nitrogen Analyzer. 1.5 g of clay was transferred to a Kjeldahl flask along with 50 mL of distilled water and 1 mL phenolphthalein.The tube was then attached to an analyser and 50% w/v NaOH n was added dropwise to the clay dispersion until a pink colour appeared. This was then distilled and the distillate was collected in a recipient with 50 mL of boric acid mixed buffer, which was titrated with 0.1 M hydrochloric acid solution. The volume of HCl used in titration was used to determine the CEC values by using the following relationship^18^:

$${\rm{CEC}}=(100\times {\rm{N}}\times {{\rm{V}}}_{{\rm{HCl}}})/{\rm{m}}$$

where,

N = Normality of standard acid (HCl)

V_HCl_ = volume of acid (HCl) used for titration

m = mass of the sample in grams

The CEC of each sample was calculated in duplicate and the average of two values was taken as a CEC (meq/100 g) of the sample.

Zeta potential charge measurement. Zeta potential charge of all the clay samples was determined with the function of pH ranging from pH 1.2–11. Sodium phosphate buffer (0.2 M) was used as the medium and pH of the buffer was measured and adjusted, if necessary, with either diluted phosphoric acid or sodium hydroxide to attain the desired pH values. The clay suspensions were prepared in respective buffer solutions and mixed for 1 hour at 60 °C followed by homogenisation for 10 min using Silverson L5M homogenizer. Zeta potential charge of all the hydrated samples was measured using Malvern Zetasizer Nano ZS. All the measurements were carried in triplicate and their mean values are reported.

Microbiological analysis. The microbial limit tests of all samples were carried out according to the US Pharmacopoeia^34^.

Total aerobic microbial count. A 1:10 dilution was prepared of each clay sample using 10 g of clay and 100 mL of phosphate buffer adjusted to pH 7.2. Further dilutions were made in a similar way as 10^2^, 10^3^, 10^4^ etc. The culture media was sterilized and cooled to 45 °C. In duplicate, 1 mL of each dilution of clay samples was poured in sterilized petri dishes. Soybean-casein digest agar medium (15 mL) was added to each plate, shaken and left to solidify. Plates were inverted and incubated for 48–72 h at 37 °C. The colonies were counted and the plates containing 30 to 300 colonies were taken into account while the other plates were rejected. The average of the counted colonies for the two plates were calculated and multiplied by the dilution factor and expressed as the number of microorganisms per gram of sample.

Test for E.coli and Salmonella species. To test for *E.coli* and *Salmonella* species, a fluid lactose medium was added to each clay sample to make 100 mL dispersion. This was incubated at 37 °C and then examined for growth. 1 mL of this pre-enriched culture was then transferred into two vessels containing, respectively, 10 ml of fluid selenite–cystine and fluid tetrathionate media, mixed, and incubated for 12–24 h at 37 °C. To test for *E.coli*, some of the fluid lactose media was streaked on the surface of MacConkey agar medium contained in the petri dishes and were then incubated at 37 °C. The dishes were then checked for the presence of the characteristic colonies of *E. coli*. Salmonella species were tested by streaking some selenite–cystine and tetrathionate media on the surface of a bismuth sulfite agar medium and a xylose–lysine–desoxycholate agar medium. The petri dishes were incubated at 37 °C for 24 h. After incubation the plates were examined for the presence of the characteristic colonies of Salmonella species.

#### Techno-economical estimation of purified montmorillonite

The costs of all chemicals and plug power meters were used for operational cost estimation of resources and power consumption, respectively.

#### Statistical analysis

Analysis of variance (ANOVA) (confidence limit of P < 0.05) was used to investigate statistical significance.

## Supplementary information

Supplementary info.
