# Supplementary material for: Exploration of a New Source of Sustainable Nanomaterial from the Koh-e-Suleiman Mountain Range of Pakistan for Industrial Applications
Source: Sci Rep. 2020 Jan 17;10:577. doi: 10.1038/s41598-020-57511-y (PMC6969096; doi:10.1038/s41598-020-57511-y)
Supplement: Supplementary file 1 — Supplementary info. [file 41598_2020_57511_MOESM1_ESM.docx]

# Exploration of a New Source of Sustainable Nanomaterial from the *Koh-e-Suleiman* Mountain Range of Pakistan for Industrial Applications

J. S. Nirwan ^1^, S. Farhaj ^1^, M. M. Chaudhary ^2^, Z. Khizer ^1^, S. S. Hasan ^1^, A. Angelis-Dimakis ^3^, A. Gill ^4^, H. Rasheed ^5^, N. Abbas ^6^, M. S. Arshad ^7^, T. Hussain ^8^, Y. Shahzad ^8^, A. M. Yousaf ^8^, T. A. Chohan ^9^, T. Hussain ^10,11^, H.A. Merchant ^1^, M. R. Akram ^12^, T. M. Khan ^9^, M. Ashraf ^5^, B. R. Conway ^1^, M. U. Ghori ^1^ *****

^1^ Department of Pharmacy, University of Huddersfield, Huddersfield, UK

^2^ Lahore Waste Management Company, Lahore, Pakistan

^3^ Department of Chemical Sciences, University of Huddersfield, Huddersfield, UK

^4^ Ministry of Minerals and Mines, Lahore, Punjab, Pakistan

^5^ Pakistan Council of research for Water Resources (PCRWR), Ministry of Science and technology, Islamabad, Pakistan

^6^ University College of Pharmacy, the University of Punjab, Lahore, Pakistan

^7^ Faculty of Pharmacy, Bahuddin Zakariya University Multan, Pakistan

^8^ Department of Pharmacy, COMSAT University Islamabad, Lahore Campus, Lahore, Pakistan.

^9^ Institute of Pharmaceutical Sciences, University of Veterinary and Animal Sciences, Lahore, Pakistan

^10^ The Wolfson Centre for Bulk Solid Handling Technology, University of Greenwich, London, UK

^11^ System Engineering Department, Military Technological College, Muscat, Oman

^12^ College of Pharmacy, University of Sargodha, Sargodha 40100, Pakistan

***Correspondence:**

Dr Muhammad Usman Ghori

**Email:** [m.ghori@hud.ac.uk](mailto:m.ghori@hud.ac.uk) ,

[muhammad.ghori@outlook.com](mailto:muhammad.ghori@outlook.com)

**Phone:** +44 (0) 1484 473295

**Fax:** +44 (0) 1484 472183

**For submission to NATURE: Scientific Reports**


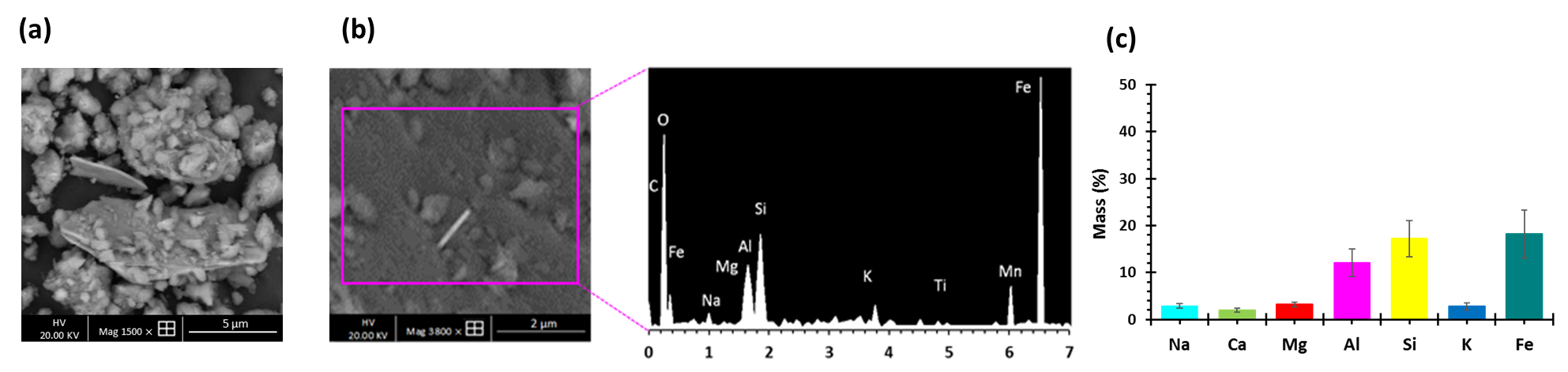


**Figure S1:** Atomic distribution of raw clay.


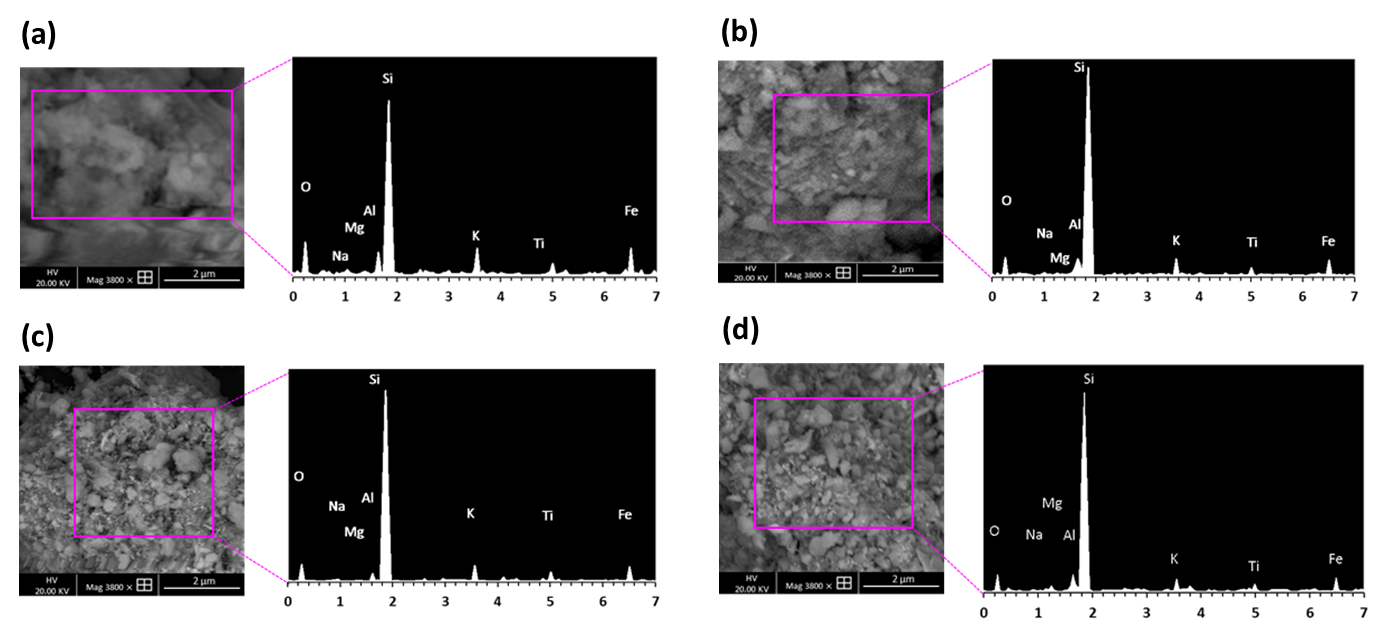


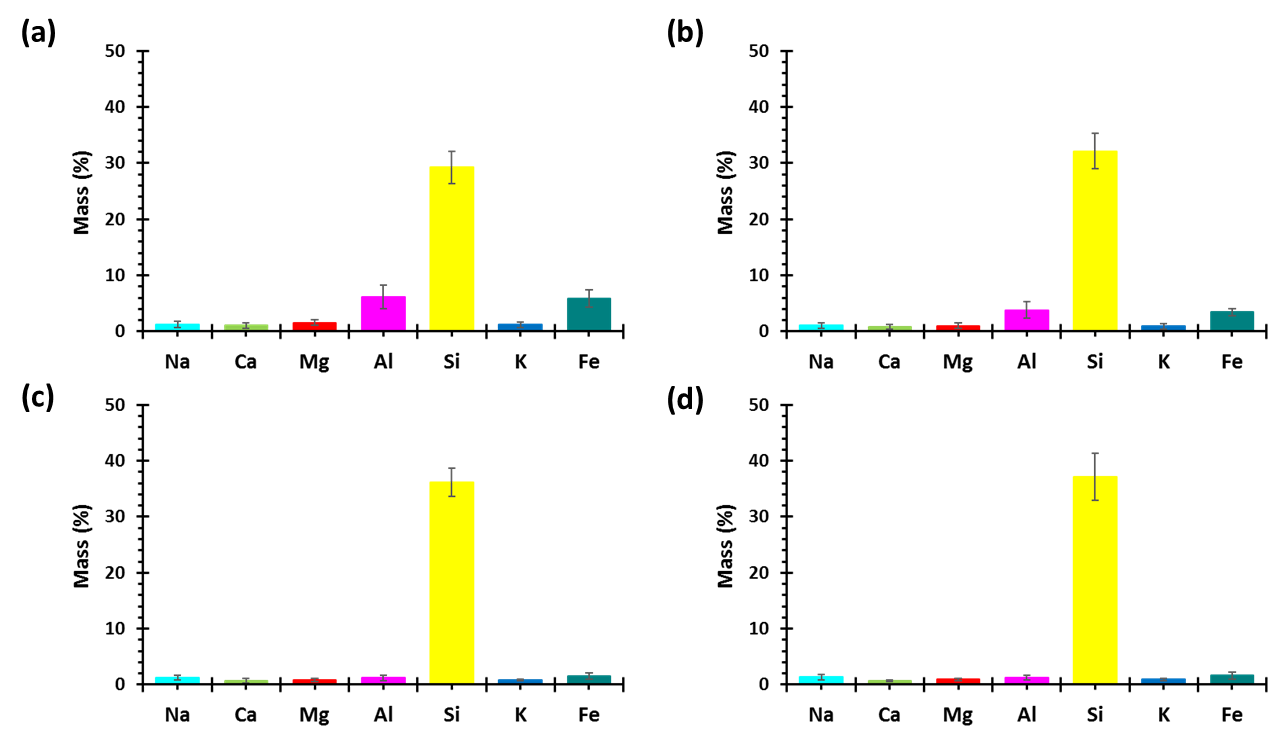


**Figure S2:** SEM micrographs with corresponding EDX analyses and atomic distribution among montmorillonite particles ground for (a) 20, (b) 40, (c) 60 and (d) 80 min.

**Figure S3:** Elementary analysis (mass %) of montmorillonite particles ground for (a) 20, (b) 40, (c) 60 and (d) 80 min.


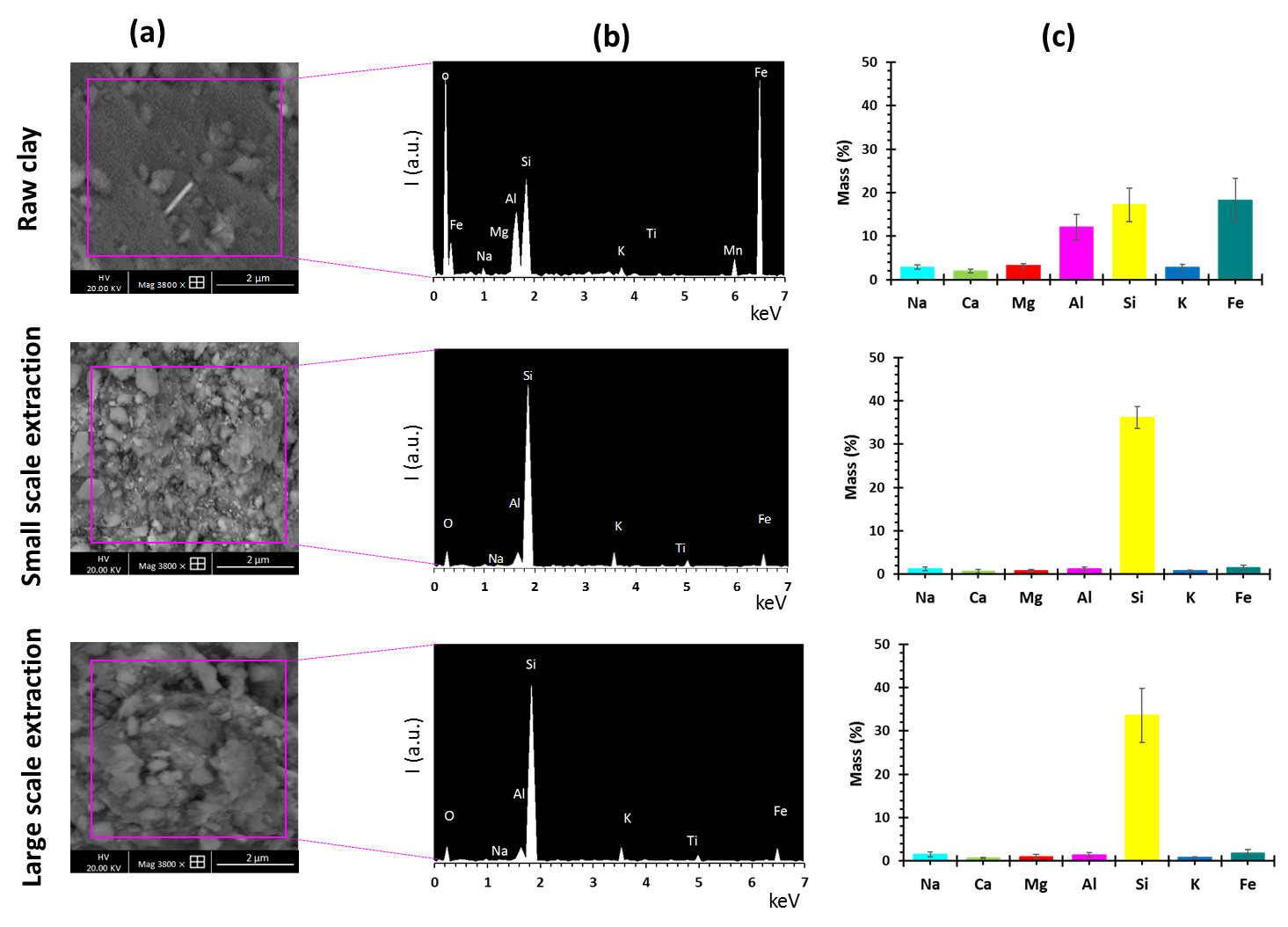


**Figure S4:** (a) SEM micrographs, (b) EDS spectra and (c) atomic distribution in clay particles
